# Clinical Notes on a Recent Series of Surgical Cases*Read at the Eleventh Annual Meeting of the Fifth District Meeting of the New York State Medical Association, Held in Brooklyn, May 28, 1895.

**Published:** 1895-06

**Authors:** Thomas H. Manley

**Affiliations:** New York; 115 West Forty-ninth St.


### CLINICAL NOTES ON A RECENT SERIES OF SURGICAL CASES.*

BY THOMAS H. MANLEY, M. D., NEW YORK.

The successful practice of surgery is not altogether unlike the cultivation of a garden; the yield will in all probability depend not so much on the richness and fertility of the soil or a favorable season, as on the knowledge, the experience, and the industry of the husbandman.

Though much in isolated instances may depend on chance and fortuitous circumstances, our chief reliance, in the end, must rest on the firm groundwork of matured knowledge and extended effort. The cases here submitted have all come under my care and observation since the first of this year and are selected because they belong to a class which commonly comes before us, and of which it may be said that now, after more than twelve years' experience in dealing with a very considerable number of them, the writer is enabled to report successes and results altogether in advance of what would have been possible at an earlier period. For time and observations have convinced me that even moderate success in the treatment of complicated surgical cases, is utterly impossible without abundant opportunities for practice, together with a familiarity with the latest and the best work of our contemporaries in our own and foreign countries.

The cases selected for presentation here are of a traumatic and pathological order, regional and general.

### CRANIAL LESIONS—FRACTURES OF THE SKULL—TRAUMA.

Since the first of March (1895), there have come under my care ten cases of fractures of the skull: four basilar, occupying the most vital, the cerebral and cerebellar regions; and six cases of fracture of the vault, the non-vital. The patients ranged in age from nine to fifty-six; there were nine males and one female; three were fatal and seven recovered. It may be said that this class of fracture is yearly becoming more com-

*Read at the Eleventh Annual Meeting of the Fifth District Meeting of the New York State Medical Association, held in Brooklyn, May 28, 1895.

mon in New York. The immediate dangers attendant on cranial fracture, arise in consequence of shock, hæmorrhage and inflammation.

The features of special interest in connection with the cases here enumerated are first, those with reference to basilar fracture involving one or more of the cranial fossæ. We had quite unequivocal symptoms of this lesion; as a free discharge of sero-sanguinolent fluid from one or both ears, with a free extravasate into the sub-conjunctional tissues.

Traumatic depression of the bone substance of the skull is not attended with such definite symptoms of sensory or motor disturbances as we would be led to expect, since the doctrine of cerebral localization has been promulgated. The most constant evidence of local injury to the cervical substances of the brain, with such aphasic symptoms as pointed to local compression, was where there were distinct marks of inward displacement of fractured bone over the fissure of Rolando, or the middle parietal convolutions.

My general line of practice in cases of fracture, through any area of the cranial vault, attended with a moderate indentation of bone, but no cerebral symptoms, is, to not interfere with a view of elevation or displacement. This line is especially adhered to when the seat of displacement is over any of the large sinuses.

*Cocaine as an Analgesic.*—In the operative technique for elevating shattered and displaced fragments, of late we have substituted pulmonary-anæsthetics altogether, by employing local cocaine analgæsia. This change has been most advantageous, to the operator, in lessening hæmorrhage, by the well-known hæmostatic action of the medicament and obviating the necessity of so many trained assistants, as would be otherwise rendered necessary.

The difference of the effects, consequent on the utilization of this line of treatment is vastly in our patient's favor; as by it there is no such engorgement of the cerebral vessels, as we always observe under ether, and no cerebral excitation, at a period when it is obvious, that violent commotion of the cerebral centres must be harmful to our patient, and persistent vomiting after operation is entirely obviated.

*Debridement, instead of the Trephine.*—Except in rare instances the trephine has been entirely displaced by me, for the osteotome in elevating and removing depressed bone fragments in skull injuries. By its cautious employment, there is much less danger of damage to the dura-mater, the brain substance, or the cranial sinuses; and less bone may be sacrificed. For simplicity, safety and readiness of employment it is a great improvement on the trephine in this class of cases.

*Asepsis and Refrigerants as Prophylactics.*—Experience has long since taught me that, except for the scalp tissues, all chemical solutions should be rigorously eschewed in all skull fractures involving an operation. My early experience soon taught me that any description of antiseptics, when applied to the nude elements of bone anywhere, the dura-mater and cerebral tissue, in particular, are full of danger to the vitality of the protoplasmic elements and may be promptly followed by grave inflammatory changes.

Now, nothing in my service is ever employed for flushing or cleansing, in cranio cephalic operations except sterilized water.

The ice-cap or iced cloths are immediately applied on the whole exposed walls of the skull, and continued until reaction is fully established.

It is my conviction, that moist cold, over a traumatized surface, is a potent prophylactic against consecutive inflammation, cellulitis or meningitis, in all skull injuries, and should be always employed, until about the third day, when it may be discontinued.

*Mercury in Meningitis.*—Mercury is an agent of unrivaled excellence, judiciously administered, in inflammatory conditions of the fibro-serous membranes. On the meninges, it acts with especial energy; therefore, in those cases of cranial trauma, a purgative dose of calomel is given early, and repeated later, should symptoms of meningeal trouble threaten; the dose of the drug being so apportioned as to promptly secure its full therapeutic effect, without its possible lethal action.

### FRACTURES OF THE LIMBS.

Fractures of the extremities, we are almost never without in the Harlem Hospital Service. In my own private hospital notes on this interesting and current type of truama, there are more than two thousand cases enumerated, though more than four thousand cases have come under my notice in the past twelve years.

Since March first this year we have had entered of fractures of the extremities, ninety-one cases; seventy-six simple, and fifteen, compound. In the deluge of surgical literature spread before the medical profession since the doctrine of antiseptics was established, it is singular to note, except that in the treatment of the compound variety, little has been presented on fractures of the extremities; in fact, this study has been allowed to lapse. There is, however, no class of surgical injuries of greater interest than those fractures which involve the structures on which we depend for locomotion and prehension. Their correct treatment will reward us with a fair degree of restoration of contour and function; while, when managed on wrong principles, diminished power and motion are quite certain to follow.

The number here includes fracture of the clavicle, arm, forearm, leg, femur and bones of the hand and foot.

*Muscular Relaxation rather than Forced Extension.*—The guiding principles which we observe in small fractures are, first, to secure muscular relaxation, and osseous reposition, without employing violence. Forced extension, or counter-extension in certain fractures, is not wanting in theoretic support; but in fracture, a considerable experience, with careful observations, have convinced me that continued tension on a limb, rather provokes muscular rigidity than effects relaxation. Besides, the co-existing pressure on the main blood-trunk, and tendency to sloughs in enforced confinement to bed are among the drawbacks of steadily maintained tension. The rules of physics will not apply to fractured bones, otherwise, such a thing as deformity after their treatment would be impossible, except in the event of great loss of bone.

Muscular relaxation with support to the mangled tissue favors the falling of the fragments into their natural position. There they will remain and restoration of contour will be the rule in many bones; but, with the clavicle and humerus, incessant respiratory movement, and in the femur, the demands of nature and the necessity of frequently shifting the body are disturbing influences which nothing can entirely overcome, and hence some sort of support is necessary.

*Support of the Fragments rather than the Fixation with Gypsum.*—When plaster of paris mold was first introduced, it was at once seized on, as the ideal material for fracture adjustment. It does serve a most useful purpose, when employed in appropriate cases; but everything is buried under it, and its removal is always attended with a jarring of the limb. For a long time it has been my custom to never employ it as a primary dressing, though after the provisional callus is formed, it answers a useful purpose.

In the treatment of *compound fractures* our first endeavor is to securely replace the fragments and close the wound—make the fracture a simple one.

In compound comminuted, with immediate, partial or complete asphyxia, or destruction of the tissues, our aim is, to first suppress all hæmorrhage by closing separately the large bleeding vessels, thoroughly cleansing and dressing the wound, and placing the parts under covering in a comfortable position.

FRACTURES OF THE SKULL (10).

| | | | |
|---|---|---|---|
| No. 10 | Vault 6. | 3 compound. | 3 single. |
| | Base 4. | All simple. | |

| | | | |
|---|---|---|---|
| Average age 32 years. | Survived | Vault 1 | Succumbed 5 |
| | | Base 2 | " 2 |

Of this number 3 were railroad injuries.
" " " 9 were males and 1 female.
Fracture of the nasal bones.
One compound fracture. Female. 36 years.

CLAVICLE (8 fractures).

| | | |
|---|---|---|
| Outer third | 7 | 5 male, 2 female, average age, 19. |
| Middle third | 1 | All simple. |

RIBS.

| | | | |
|---|---|---|---|
| 5th rib (1) | 6th rib (1) | 8th rib (1) | All simple. |
| 9th rib (2) | 7th rib (2) | 10th rib (2) | |

HUMERUS (13).

| | | |
|---|---|---|
| Surgical neck | (1) | Simple. |
| Middle third | (6) | 1 compound. |
| Lower third | (2) | 1 compound. |
| Extern. condyle | (1) | Simple. |
| Internal condyle | (3) | Simple. |

RADIUS (18).

| | | | | |
|---|---|---|---|---|
| Middle third | (2) | 1 compound. | | |
| Colles' fracture | (16) | Simple. | 6 female. | 7 male. |

ULNA (2).

| | | |
|---|---|---|
| Middle Third | (2) | Simple. |

METACARPAL BONES (1).

Metacarpal bone of index finger.

PHALANGES OF HAND (6).

2d phalanx. Index and middle finger.
1st and 2d phalanges. Thumb.
1st and 2d phalanges { Index and middle fingers. Compound fractures.
All phalanges of right hand. Compound.
2d phalange. Ring finger. Simple. No amputation.
3d phalange. Little finger. Traumatic amputation.

FEMUR (6).

| | | |
|---|---|---|
| Intracapsular | (3) | Simple. |
| Middle Third | (2) | |
| Supracondyloid | (1) | Compound. Railroad. |

TIBIA (2).

| | | |
|---|---|---|
| Middle third | (7) | 5 compound. |
| Lower third | (3) | 1 Traumatic amputation. |

TIBULA (15).

| | | |
|---|---|---|
| Middle third | (7) | 2 compound. Simple. |
| Palb's fracture | (8) | All simple. |

PATELLA (1).

Nasal Bones (1) Compound.

TARSAL BONES (1).

1 railroad case, in which all the bones of the foot were ground into pulp.

METATARSAL BONES. (3)

3rd metatarsal.
1st and 2d metatarsal.
All metatarsal bones. Compound. (Railroad.)

PHALANGES (OF FOOT).

1st and 2d phalanges of 1st and 2d toes. } Compound.

1st and 2d phalanges of 4th and 5th toes. }

Little toe. Traumatic amputation-

*PRIMARY AMPUTATIONS.*

Primary amputations after disorganization of a limb in civil life, is always questionable procedure, though when a limb has been practically traumatically amputated, we may at once with the scissors, divide the frayed integument or tendons. There have been lately eight amputations in my service, for traumatism and pathological conditions. After reaction when the line of demarkation is formed in traumatic cases we run little risk of a sloughing flap.

Such a case is now under my care. The young man had his foot crushed off at the ankle joint, by a railroad accident. Four days later, after the line dividing the healthy from the dead tissue appeared, sufficient of the parts under the healthy skin were cut away to allow the healthy integument to fal in over the ends of the bones and cover them without strain.

CERVICAL TUMORS.

Ten cases of serious submaxillary cervical tumors have come under my care within the past three months.

One was a case of sarcoma of large size, in an old man of 64; was refused operation. It was not apparently deeply lodged, though as there were rapidly developing new growths in the hard-palate, pharynx and sphenopalatine fossa, it was decided that an operation was not warranted.

One tumor of large size was a lipoma; this sprang from the loose subscripital tissues and advanced forward and downward to dip under the posterior of the sterno-mastoid muscle.

*Importance of Accurate Diagnosis.*—One man on whom we operated had a voluminous growth, which occupied the whole quadrangle of the neck; on right side advancing up under the lower jaw and downward behind the left clavicle to and through the apex of the pleural cavity. He had been operated on four months previously, the growth appearing consecutively, rapidly enlarging and now, so far encroaching forward under the deep cervical fascia as to encroach on the trachea-rings and threaten suffocation.

At the institution in which he was first treated, several operations were refused, as the growth was declared malignant. The mass was hard, unyielding and painful, with deep attachments. On a careful and thorough examination of his case, it was my conclusion that the mass was wholly tubercular, and that, while its removal would demand a very formidable operation, it was clearly within the range of operative surgery to turn it out safely. It was presumed that in the first operation the superficial absorbents were removed, and that the re-growth consisted in a hyperplasia, with central degeneration of the deep chain, which lies in deep contact with structures vital to life.

*Hemorrhages in Operations on Voluminous Cervical Tumors for their Removal.*—The most formidable danger in the complete removal of cervical neoplasms, comes from hæmorrhage; and there is nothing that so severely tries our courage and skill, as the contact with it, in this class of cases. Celerity and coolness must be combined when an arterial spring is opened, or the operative field is suddenly flooded, by the vascular torrent. In infiltrating malignant growths of the neck their removal always entails great danger, because of a steady oozing which cannot always be wholly staunched before dangerous anæmia sets in.

In this case, the operation of enucleation was commenced by dividing all the overlying structures in the centre, a cervical incision having extended across the entire mass. Great care was taken to clamp and ligate all the bleeding vessels as we proceeded. The peripheral decorticature was comparatively simple; but as we penetrated into deep parts, difficulties began to appear.

Early in the operation the internaljugular vein was divided, and both ends ligated. Next, as we proceeded more deeply to turn the growth out consecutively, the cervicalisascendens, the ascending thyroid, the transversaliscoli and occipital arteries were clamped, ligated and divided. Of thenerves the descendimoni phrenic and anterior cervical branches of the cervical-plexus were divided and their ends leased away from the elements of the tumor. The common carotid was adherent by its sheath, from the clavicle, up to the point of bifurcation. The sheath with the pulsating vessel was cautiously detached from the under surface of the growth, and now, while endeavoring to enucleate the deep supra-clavicular lymphatics and press forward the subclavian artery, the pleural cavity was opened, just below where the subclavian arches upwards. Now, the whole mass was lifted out, leaving a large chasm, exposing the tracheal plexus, the large blood-trunks and trachea.

The opened pleura was then closed, the bleeding subsided and the edges of the integument were brought together. In this case, although there was an extensive mutilation of tissue, but little blood had been lost. Our patient rallied well from the operation. His recovery was prompt, the line of incision closing by primary union, except at the part left for drainage. But little blood had been lost and no shock followed.

The mass proved to consist of an aggregation of tubercular glands; some simply hypertrophical, others were suppuratory and caseous; all bound together by a dense inelastic matting of fibro-connective tissue. Although the thyroid submaxillary and sublingual glands were freely exposed, no trace of infiltration was visible.

Of the other seven cases, two were malignant, two were acute suppurative inflammation of the lymphatics; the remaining three, evidently two, were tubercular, but not suppurating. These are being treated by palliative measures.

HERNIA—VARIOUS TYPES.

Sixteen cases of various types of hernia have come under my notice since the beginning of the present term; these included strangulated, incarceration, reducible and irreducible cases. But one case required operation for strangulation. The operation was performed late at night, by a member of the House-Staff.

*Death in Strangulation Attributable to Delay and Avoidable Errors in Operating.*—This case, which terminated mortally, was most fruitful in suggestion and in pointing the way to avoiding mistakes in treatment. The case was neglected in the beginning by the practitioner who was first called, by persisting in violent taxis and allowing his patient to suffer until collapse set in, before sending for or calling in active surgical relief.

The next oversight was committed in the details of operation. A knuckle of intestine partly twisted on itself, with a patch of gangrene, about the size of a quarter, extended through the glandular and muscular tunics and occupied the walls of the bowel; though there was no perforation, as the outer fibro-cellular coat was intact. Vomiting ceased after the constriction was relieved and the intestine returned; but the man did not react and sank thirty hours after operation. On autopsy it was found that a fatal error had been committed in the management of the extended coil. The adhesion which held the coiled, bent surfaces of the intestine together, had not been liberated and, in consequence, its lumen remaining completely obstructed, internal strangulation followed, thus removing every possible hope of recovery. The gangrenous area in my opinion was not enough to seriously call for resection, the use of the Murphy anastomotic button or other adjustment, inasmuch as it extended only about half way over the entire cylinder and would undoubtedly have taken care of itself by taking on adhesion to some of the neighboring viscera, thereby preventing any possible leakage.

*Critical Inspection of the Protruding Mass and Thorough Liberation of all Constriction, both within the Sac, the Canal and at the Internal Ring, Indispensable, in all Cases of Operation for Strangulation.*—In all cases of operation for strangulation, after we have freely divided the points of stenotic impediment, it is of the greatest importance that we critically inspect the conditions of the viscera and completely liberate the imprisoned structures. A failure to do this, renders an otherwise life-saving operation inert in its effects. A practical demonstration of this came under my notice recently. A man was seized with symptoms of strangulation after stool. He had a chronic incarcerated inguinal epiplocele. An incision was made down in the upper surface of the mass, the omentum exposed and the inner ring divided. But there was no relief of the symptoms after operation and the patient sank unrelieved the following day. On autopsy, a loop of intestine was found caught, crushed under and concealed by the omentum. This had entirely escaped detection and continued occluded after the epiplocele was exposed.

*Treatment of Reducible Hernia.*—Nine cases of reducible inguinal hernia have been examined by me, within the past ten weeks. My practice in this class of cases, is, not to advise operation, unless the hernia is progressively enlarging, is painful, threatens strangulation, is not trussable, or the patient is desirous of relief from the deformity. Besides the class enumerated, it is my custom to recommend a radical cure in a female or in those individuals whose deformity constitutes an impediment to entrance into the civil or military service. But one of these nine which came to me was selected as appropriate for radical cure.

He was a young man who had hernia since childhood. It was of the indirect inguinal type. This was operated on with gratifying results, the patient leaving the hospital in two weeks.

*O'Hara's or the Australian Operation the best for Open Treatment.*—Of the almost infinite number of operations recommended during the past ten years for cure of hernia, there is none which in so large a measure fulfills the requirements with so little mutilation, as the operation devised by Mr. Henry M. O'Hara, of Melbourne, Australia. Briefly described it consists in an isolation of the neck of the sac, its division and retraction up through the canal and internal ring, to the fascia transversalis, where it is anchored by absorbable suture material. It entails no weakening of the abdominal walls, by divisions of the aponeurosis, no divisions of large vessels; there is no drainage required and no disfiguring scars left. In fifteen cases so treated by me since April, 1894, there have been no relapses.

### ABDOMINAL SURGERY.

Without doubt in the whole field of medical science and since its earliest dawn there has never been anything to equal the stupendous progress made in *abdominal and pelvic* surgery during the past twenty years, and in this (let medical history record it) America has occupied the position of the pioneer. In 1881, the celebrated Simms gave to the professional world his conceptions on the possibilities of surgery in this direction; though unhappily he did not survive to realize the fruition of his hopes. Biglow had perfected litholosity; at the International Medical Congress in Washington in 1886, Nicholas Senn presented his thesis on the surgery of the intestine and startled the rank and file of the profession, by the immensity of his original and marvelous researches and experimentation on abdominal surgery in the lower animal, and his lucid demonstration of their application on man. Abbe had been a generous contributor in this line and posterity is indebted to Sands for opening the way to that operation on the appendix, since perfected by McBurney; and through which, thousands of lives have been saved. It has rendered necessary the re-writing of the pathology of the peritoneum. As would seem the climax of all, the young Chicago surgeon, Murphy, has invented an anastomotic button, the very acme of human ingenuity, which has rendered the surgery of the intestine more expedient and safer than ever.

### APPENDICITIS.

*Timeliness and Skill in Operation on the Appeudix.*—During one week from April 5th to 13th, in this year, seven cases of severe abdominal disease came under my notice. Six were of appendicitis, four were of the perforating type. Two came into the hospital, in the advanced stages, too late for relief by operation and both died within twenty-four hours after entrance. Two were operated on, with perforation of the appendix in both cases; in one, there was a double perforation with general peritonitis; both recovered. Two mild cases of the recurrent type were treated by palliative measures, and recovered. In one abdominal case, which was brought into the hospital, great prostration was present. My colleague, Dr. A. Palmer Dudley, was of the opinion that there was mechanical obstruction; though it was my impression that it was the appendix which was at fault and that obstruction was due to intestinal paresis. The abdomen was so extremely tympanitic that it was impossible to definitely locate the structures. On section the appendix was found healthy and as Dr. Dudley had predicted, the small intestine was found obstructed by a band at about its center. The patient rapidly sank under ether, before the incision could be closed.

It was formerly my conviction that as the danger in operation itself seemed to me very great, and the larger part of those cases of appendicitis do well under medical treatment alone, surgical interference was not proper in any except rare instances. Later experience has convinced me that this position is not rational, and that in all cases of appendicitis which show a tendency to persistently recur or are of the acute fulminant type, the correct course is prompt operation. The results in operation depend, almost entirely, on operating at just the right time and performing the operation with skill and celerity. The time to operate is, and always probably will be, very difficult to determine. Its performance for a successful issue in a large number requires special skill and experience. A small incision, the quick ferreting out of the appendix under the trained finger, the neat and aseptic amputation of it, without the loss of unnecessary time or trauma of the peritoneal, count vastly in the patient's favor.

On the 28th of April, Dr. J. A. Hoffheimer called me in consultation to see a case of appendicitis under his charge with the view of determining the propriety of an operation.

The patient was a boy of twelve, who had had previous attacks. It was the sixth day of his illness, when I saw the case.

At this time, his pulse was 143 per minute, the temperature 103, there was some vomiting and severe pain, when morphine was not pressed. The abdomen was extremely tympanitic and over the appendix sensation to pain on pressure was extremely acute, though all the abdominal areas were hyperæsthetic.

After a careful examination of the case, giving due weight to the various serious symptoms present, the lad's grave condition, and the uncertainty of results after radical measures when the advanced stage of the disease is reached, my opinion was, that nothing definitely could be promised by operation, and that it was possible that recovery might ensue by energetic constitutional measures. Three days later the boy died just at a time when it seemed that peritoneal inflammation had subsided. Now my regret is, that I did not operate. Indeed, there can be scarcely a question but in all these cases, no matter what the stages of inflammation may be when we are once assured that the appendix is the actual seat of serious disease, an immediate operation should be invariably insisted on.

### JOINT DISEASES.

*The Influence of Systemic Cachexia on the Arthrop-athies which Succeed Injuries.*—Several cases illustrating the various phases of joint *injuries* and *diseases* have lately come into the hospital for treatment. The dominating pathological changes attending these were rheumatism and tuberculosis. There can be scarcely a doubt but various constitutional disturbances impress their stamp on many traumatisms and enhance the vulnerability of various organs and structures. This is especially true of the tissues which enter into the joints.

The articular heads of the bones are the center of great activity during the processes of growth and development. As a move in the direction of arriving at an accurate knowledge of the precise pathological condition which obtains, in certain arthritic elements it would be well if the term "hip-joint disease or "knee-joint disease" were abolished.

A young man, in late autumn, 1894, sustained a sprain of the knee, in a football game. He was treated at home and elsewhere for "knee-joint disease." Failing to secure relief from the various fixation appliances which had been employed, he entered Harlem Hospital on the 12th of March. On a critical examination of the joint there was no evidence of any morbid changes in the capsules or cartilages; but the head of his tibia was enlarged and exquisitely sensitive. The cancellous center of this was opened freely; the trephine entering just under the insertion of the tendon of the sartorious. A pus cavity was tapped and a considerable residue of necrosed bone curetted out. All pain immediately ceased, the spasmodic contraction of the hamstrings passed off. He returned in four weeks to his trade of a painter when his "knee-joint disease," (?) was cured. A young man entered later, who a few days before had violently wrenched his knee. He was sent in to have the supposed hæmorrhagic distension of the capsule relieved by evacuation through an ortheotomy.

It was found to consist entirely of a hæmatoma into the loose cellular tissue outside the aponeurotic investment of the knee joint and wholly disappeared by a few days rest, with moderate bandage-pressure.

A young woman of good physique was sent into the hospital for the treatment of "acute suppurative synovitis of the right knee-joint." On inspection it was found that the patella was displaced inward and deeply lodged under the projecting surface of the voluminous fullness, coming down from above. The case was clearly one of acute phlegmon under the fascia-lata; its fluid contents being arrested from advancing further downward, by the insertion of its fibrous hood into the lateral surfaces of the quadriceps tendon. The articulation had wholly escaped. An incision, evacuation and drainage gave immediate relief and final recovery was rapid.

*Rheumatic Pain Sometimes Antecedent to Trauma of the Joints.*— Rheumatic affections of the joints of the lower extremities are sometimes preceded by a weakening of the muscles. This precedes the intense pain and swelling. The person about to be seized is conscious of a sense of lameness or unsteadiness in the articulation. He is now liable to a fall or wrench, when all of a sudden, all the typical symptoms of acute inflammation set in and he has a pan-arthritis. To mistake this mixed condition for a joint trauma alone, and concentrate all one's attention on the local trouble may lead to serious results. Protracted fixation of a limb always interferes with its full nutrition and arrests its growth in growing children.

Several cases seen by me, during the spring months, supposed to be organic disease of the joints, promptly recovered when all retaining supports were removed, the joints allowed full liberty and rheumatic remedies pressed.

*Resection of Diseased Joints or Conservative Methods.*—Formal resection of a joint is never done in my service, unless there is evidence that disease has completely disorganized it. In those cases it remains a question if an amputation is not preferable. Resection of a joint, let it be remembered, means its entire destruction. In the child, under ordinary surroundings, after a time, tubercular disease of the heads of bone often tends to spontaneous arrest. My practice in aggravated cases of this type is to open and curette the joint, preserving the ligament and cartilages. In adult tuberculosis of bone, reaction will not arrest the progress of the malady which is now usually generalized and progressive.

### MAMMARY TUMORS.

Among the cases seen and treated by me within the past quarter, were five cases of tumor of the mammary gland. Three of them were malignant, of the epithelial variety, one recurrent. Of the other two, one was a dermoid cyst and the other tubercular.

*The Therapy of Mammary Neoplasmata.*—The cyst was readily decorticated, the incision promptly healing. Seven years before she had the opposite breast removed for the same condition in which, up to the present time, there had been no recurrence. An incision, grattage and drainage cleared away the strumous infiltrate.

The principles which should govern us in the management of cancer of the breast are still *sub judice*, indefinite and unsettled, although the reports coming in from the results of so many tumors, extirpation of all the axillary absorbents, with the complete removal of the mammary gland, point to a great improvement of this method, above all others.

One of three cases was treated by me in this plan for the reason that there was already an immense secondary growth in the axilla. In her case there was a large, hard, infiltrating, secondary growth in the axillary hollow, which required a very delicate dissection to remove it without damage to the main artery, to the trunk of which the tumor maintained a firm grip.

The long thoracic artery with the thoracic alarix were divided and the axillary vein opened. But all hæmorrhage was readily subdued. The patient ultimately made a good recovery, though with marked limitation of shoulder action, in consequence of the cicatrical construction in the apex of the axillary space. The direction of dissemination in schirrus is always centripetal along the course of the absorbent vessels. The adenomatous structnres of the lymphatics, serve as outposts to prevent systemic infection, which no doubt are invaded at an early date. The complete operation is one which entails an extensive mutilation of tissue and is perhaps more dangerous to life than simple mammary excision; but, the promise which it gives us against relapse more than outweighs these objections.

One patient with a small, hard schirrus refused operation and later, I was informed, went into the hands of a charlatan.

One case of recurrent cancer had been first operated on by me in November, 1884, and again six months later. She had refused any further cutting operation.

In her case the powerful chemical cauteries have been employed by me, with the hope of being able to clear away the fungating crop of pulpy granulations, which occupied that site of the scar tissue. It succeeded fairly well at first, but its repetition in an attempt to destroy the substrata of neoplastic elements, was attended by such agonizing pain and positive shock that life was endangered, and my patient declared she would rather die than undergo the ordeal again.

When arsenical paste was employed although its activity in charring the tissues was decided, yet there were invariably symptoms of constitutional poisoning; numbness of the extremities from toxine, neumitis, nephritic irritation with sore throat and very severe gastric symptoms followed in every instance.

My own unsatisfactory experience with caustics has led me to employ them in malignant growths with caution, and never, except on those which occupy the periphery and are of a very limited area. In labial epithelioma or on senile growth involving the nose or eyelids, the caustic substance will serve a most useful purpose.

The above incomplete notes and comments are on a class of cases that commonly come under our observation and of late years have been treated with such success as was heretofore quite impossible. If in only a limited degree I have succeeded in calling attention to some of their most salient features and indicated rational methods in their management, my aim has been accomplished.

115 *West Forty-ninth St.*

